# Slouching towards engagement: interactions between people using psychedelics naturalistically and their healthcare providers

**DOI:** 10.3389/fpsyt.2023.1224551

**Published:** 2023-08-04

**Authors:** Kevin F. Boehnke, Kasey Cox, Cody Weston, Moss Herberholz, Nicolas Glynos, Nicholas Kolbman, Christopher W. Fields, Julie Barron, Daniel J. Kruger

**Affiliations:** ^1^Anesthesiology Department, University of Michigan Medical School, Ann Arbor, MI, United States; ^2^Chronic Pain and Fatigue Research Center, University of Michigan Medical School, Ann Arbor, MI, United States; ^3^Michigan Psychedelic Center, University of Michigan, Ann Arbor, MI, United States; ^4^Psychiatry Department, University of Michigan Medical School, Ann Arbor, MI, United States; ^5^The Radical Well-Being Center, Southfield, MI, United States; ^6^Blue Sage Health Consulting, Ann Arbor, MI, United States; ^7^Michigan Psychedelic Society, Ann Arbor, MI, United States; ^8^Decriminalize Nature Ann Arbor, Ann Arbor, MI, United States; ^9^Population Studies Center, University of Michigan, Ann Arbor, MI, United States

**Keywords:** psychedelics, psychiatrist, antidepressants, mental health, psilocybin, primary care physician

## Abstract

**Introduction:**

There is substantial public interest in psychedelics as potential treatments for psychiatric conditions. However, most psychedelics are criminalized under federal law in the USA, so it is unclear whether use occurs with clinical support. Our objective was to assess whether naturalistic psychedelic use occurs with clinical support, interactions between those using psychedelics and healthcare providers (psychiatrist, therapist, or primary physicians), and use characteristics.

**Methods:**

We conducted an online, anonymous, confidential, cross-sectional survey of adults reporting psychedelic use (*N* = 1221) through a psychedelics advocacy event and social media between 9/18/2022 and 11/5/2022. We assessed participant disclosure of psychedelic use with their psychiatric care provider (PsyCP) and/or primary care provider (PCP), desire for provider support, access to support, and rate of taking prescribed psychoactive medications alongside psychedelics.

**Results:**

Among participants with such care providers, 22% disclosed psychedelic use to their PCP vs. 58% to their PsyCP. Participants were less confident in PCP vs. PsyCP ability to integrate psychedelics into treatment. Common reasons for nondisclosure included stigma, inadequate provider knowledge, and legal concerns. 23% reported taking psychedelics on the same day as potentially interacting psychiatric medications (e.g., anxiolytics, antidepressants). Despite 81% of participants desiring therapist support during psychedelic experiences, only 15% had received such support.

**Discussion:**

Our results show that psychedelic use is generally disconnected from primary and psychiatric clinical care. This disconnection may result in safety issues, including inadequate screening for contraindicated conditions, lack of support during emergent adverse events, and drug interactions. Enhanced clinical education and orienting drug policy towards known harms and benefits of psychedelics is needed.

## 1. Introduction

The field of psychedelic research has grown rapidly in the past decade, in part because of a changing political landscape and an overall desire for effective, alternative pharmacologic therapies for psychiatric conditions (1). Clinical trials have demonstrated a promising future for psychedelic-assisted psychotherapy, and both healthcare providers and the general public demonstrate an interest in the potential of these therapies (2, 3). Indeed, psilocybin administration, in concert with professional therapeutic support, has shown promising preliminary results for the treatment of major depression and treatment resistant depression (4–6), depression and anxiety in patients with life-threatening cancer (7–9), as well as alcohol and tobacco dependence (10–12). Antidepressant and anxiolytic effects as well as improvements in post-traumatic stress disorder symptoms have also been reported following use of other psychedelics including MDMA (3,4-methylenedioxymethamphetamine) (13, 14), the *N,N*-Dimethyltryptamine (DMT)-containing beverage ayahuasca (15, 16), ketamine (17), and lysergic acid diethylamide (LSD) (18). However, these compounds are not without risks: despite possible systematic underreporting of adverse events in clinical trials, there have been several known cases of serious adverse events such as suicidal ideation (5). Further, the psychological vulnerability and suggestibility elicited by psychedelics requires thoughtful care and support when used in the context of therapeutic relationships, as displayed by documented cases of abuse by psychedelic therapists, even in clinical trial settings (19, 20).

New legislation in Oregon, Colorado, and numerous municipalities in the U.S. has begun to decriminalize use of psychedelics, and national trends show an uptick in self-administered naturalistic psychedelic use (1, 21). These laws typically remove criminal penalties for possession of psychedelics but do not explicitly enable a commercial marketplace. While Oregon and Colorado legislation included provisions to allow psilocybin-assisted therapy treatment centers under providers with specific training, Oregon’s program just opened and Colorado’s is still under development. Elsewhere, naturalistic use typically occurs without healthcare provider oversight and is often for mental health reasons. For example, we recently conducted an online survey of 1,435 individuals, over half of whom used psychedelics for psychiatric conditions, most commonly anxiety and depression. In this survey, over 70% of participants never discussed their psychedelic use with their primary healthcare professional, although ~80% of participants said they would be very or extremely likely to seek support from a therapeutic provider for their psychedelic experience if one were legal and available (22). The main reasons for not disclosing use were due to stigma, legal concerns, and perceptions that their healthcare providers would be unable to successfully integrate psychedelics into their treatment. Although understandable in the current legal climate, this lack of communication raises concerns as there are several potential risks of unmonitored use, especially in individuals with conditions such as psychotic or bipolar disorders, post-traumatic stress disorder, or valvular heart disease, as well as among those taking medications that may interact with psychedelics, such as antidepressants or anxiolytics (23, 24).

Many individuals in this previous report also used psychedelics for psychiatric conditions – most commonly anxiety and depression (22). Unfortunately, there is minimal literature on the interactions between mental health providers and patients using psychedelics naturalistically. A recent survey of US-based clinical psychologists reported that nearly ⅔ of respondents did not have a clear understanding of psychedelic effects and would need to seek additional support to work with patients using psychedelics (2). Similarly, a 2021 survey of psychiatrists highlighted concerns about the lack of trained providers available for implementing psychedelic-based treatments (3). However, we are unaware of any reports documenting the other side of the therapeutic relationship – i.e., patient perspectives on interactions with psychiatric health providers about psychedelics.

Thus, in this report we explore the perceptions and preferences of individuals regarding psychedelic use in the context of their own general and mental healthcare. Specifically, we explore participants’ overall desire for provider support, access to support, preferences for professional versus underground settings, and their decisions to disclose psychedelic use with their psychiatric care providers (PsyCP) and/or primary care provider (PCP). We also investigated patient-reported combinations of taking prescribed psychoactive medications alongside psychedelics. As most therapeutic psychedelic research has explored effects on mental health conditions, we hypothesized that individuals would be more likely to discuss naturalistic use of psychedelics in the setting of psychiatric care when compared to primary care. We anticipate, however, that patients have low confidence in their providers’ knowledge about and ability to integrate psychedelics into their treatment, even though there is an overall desire for the support of trained providers.

## 2. Methods

### 2.1. Participant recruitment and eligibility

We recruited respondents for this survey study via a confidential, anonymous survey link (Qualtrics, Provo, UT) that was open from September 18th to November 5th, 2022. Study recruitment occurred in two venues: (1) in person and via posted advertisements at a psychedelic advocacy event in Ann Arbor, MI and (2) online via email listservs and social media platforms such as Facebook and Reddit. We enabled the “Prevent Ballot Box Stuffing” setting in Qualtrics to prevent duplicate responses. To participate, individuals needed to be >18 years, report past psychedelic use, and live in the United States.

### 2.2. Survey development

As described previously, we developed this survey collaboratively with input from various stakeholders, including therapists, psychedelic advocacy groups, and academics experienced in survey design and psychedelic research (25). The current study reports on components of this survey that investigated psychedelic use patterns as well as interactions related to psychedelics with healthcare providers, including PCPs and PsyCPs. We collected sociodemographic information and any past medical diagnoses of physical (e.g., cancer, diabetes) or mental health conditions (e.g., depression, anxiety, addiction), as well as information on psychedelic consumption (frequency of consumption, types of psychedelics consumed).

### 2.3. Measures

In addition to domains described above, we assessed how participants interacted with their PCP about psychedelic use. These included questions about whether participants had disclosed psychedelic use to their PCP, and their confidence in their PCP’s ability to integrate psychedelics into a treatment plan. Confidence level was assessed via a 5-point Likert scale, which ranged from “Not at all” to “Completely” confident. Those who did not disclose psychedelic use to their PCP were asked for rationale of non-disclosure, with options including privacy, legal concerns, stigma, and other choices.

Given that most naturalistic use of psychedelics was for mental health reasons (22), we asked participants about their interactions with PsyCPs. We used a similar battery of questions to those described above, with further questions about whether and what kind of psychiatric medications participants took or had taken on the same day as using psychedelics, as some medications may be contraindicated due to safety concerns and/or may blunt psychedelic effects (e.g., benzodiazepines, SSRIs) (26, 27). Participants who disclosed taking psychiatric medications on the same day as psychedelics were asked whether they believed there were any risks of combining these medications with psychedelics.

We asked participants about whether they had preparatory support before, during, and/or after psychedelic experiences, their likelihood of using psychedelics with a therapeutic provider (licensed or underground) and whether they had consumed psychedelics with a trained therapist or healthcare professional. Finally, participants were asked whether they thought that therapists and healthcare providers needed to experience psychedelics themselves in order to guide others, with responses on 5-point Likert scale questions ranging from “strongly disagree” to “strongly agree.”

A full list of the survey questions is provided in the appendix.

### 2.4. Analyses

We characterized the study sample using descriptive statistics. Categorical variables are reported as *n* (%), and continuous variables are reported as mean ± SD and range. Measures with Likert scales (e.g., how confident participants were in their healthcare provider’s ability to integrate cannabis into treatment) were analyzed continuously. Using independent sample *t*-tests (effect sizes calculated using Cohen’s *d* statistic), we assessed whether participants who had disclosed psychedelic use to their PCP or PsyCP reported differences in their perceptions of whether they had adequate preparation for, support during, and support following psychedelic experiences. All analyses were conducted via SPSS version 27 (IBM Corp. 2020).

### 2.5. Ethics statement

All surveys and procedures were approved by University of Michigan Health and Behavioral Sciences Institutional Review Board under the protocol HUM00205639 prior to data collection. Participants could withdraw at any time and were not compensated. Snacks were available at the advocacy event recruitment table to attract participant attention and interest.

## 3. Results

Overall, 1,287 of 2,151 initiated surveys were completed (59.8% completion). Of these, *n* = 66 participants reported never taking psychedelics, leaving a final study sample of *N* = 1,221 individuals. These participants were 48% women, largely white (85%), 39 ± 12 years old on average, and 59% had a bachelor’s degree or higher level of education (Table 1). Only *n* = 17 (1.4%) lived in Oregon, the only state with decriminalized psychedelics at the time the survey was live. Most (67.2%) participants used psychedelics twice a year or more frequently, 23.7% used psychedelics once a month or more often. About one-fifth (18.2%) of participants had only used psychedelics once or had not used psychedelics in the past 5 years. The most used psychedelics were psilocybin mushrooms (93.4%), LSD (66.6%), and MDMA/MDA (57.0%). Smaller proportions of participants had used substances such as Ketamine (32.0%), DMT (30.5%), *Salvia divinorum* or salvinorin A (25.3%), mescaline (19.5%), other synthetic phenethylamines (14.1%), or ayahuasca (13.7%). Participants used an average of three different psychedelics (SD = 3), and 23.9% had used six or more different psychedelics. Most (85.7%) had used moderate or high doses of psychedelics while 14.3% had only used microdoses.

**Table 1:** Participant sociodemographics.

Descriptive	
Gender (*n* = 1,214)	
Women	48%
Men	47%
Non-Binary	2%
Transgender	<1%
Other	2%
Age in years (M, SD, range)	39, 12, 18–84
Education in years (*n* = 1,214; M, SD, range)	16, 2, 10–20
High school graduate, GED, or less	7%
Some college, technical, or associate’s degree	34%
Bachelor’s Degree	32%
Master’s, Doctorate or Professional Degree	27%
Annual household income (*n* = 1,186)	
$50,000 or less	36%
$50,001–$100,000	31%
$100,001–$150,000	17%
More than $150,000	16%
Races/ethnicities (inclusive; *n* = 1,170)	
White	85%
Hispanic/Latino	6%
Native American	4%
African American/Black	3%
Asian	3%
Native Hawaiian/Pacific Islander	<1%
Other	5%

The most common past diagnoses cited by participants were depression (62%), anxiety (52%), and attention deficit hyperactivity disorder (ADHD, 27%). Overall, 22% of participants had discussed their psychedelic use with their PCP, while among the subset of *n* = 274 participants who reported having a PsyCP, 58% had discussed their psychedelic use with this PsyCP (Table 2). Compared to PCPs, participants were significantly more confident in their PsyCP’s ability to integrate psychedelics into treatment (mean difference = 0.66, 95% CI: 0.46–0.87, *p* < 0.001). The most common rationale for not disclosing psychedelic use to either PCPs or PsyCPs was stigma (59% for PCP and 70% for PsyCP).

**Table 2:** Interactions with healthcare providers.

	Primary care provider (PCP) *n* (%)	Psychiatric care provider (PsyCP) *n* (%)
Has care provider?***		
Yes	1,103 (90%)	274 (22%)
Discussed psychedelics with care provider?***		
Yes	239 (22%)	160 (58%)
No	864 (78%)	114 (42%)
Reasons why psychedelics not discussed		
Concern about stigma associated with psychedelic use*	509 (59%)	80 (70%)
My care provider is not adequately knowledgeable about psychedelics*	422 (49%)	44 (38%)
Legal concerns	361 (42%)	39 (34%)
I prefer to keep my psychedelic use private**	343 (40%)	31 (28%)
No reason to discuss***	337 (40%)	26 (24%)
I do not trust my care provider	173 (20%)	26 (23%)
Other	53 (6%)	10 (7%)
Confidence in care provider’s ability to integrate psychedelics into treatment***		
Completely confident	15 (1%)	29 (11%)
Very confident	24 (2%)	22 (8%)
Moderately confident	69 (6%)	36 (13%)
Somewhat	138 (13%)	49 (18%)
Not at all or somewhat	733 (67%)	112 (41%)
Do not know	123 (11%)	26 (9%)

*p < 0.05, **p < 0.01, and ***p < 0.001.

Overall, 23% of participants reported taking psychiatric medications on the day of their psychedelic experience (Table 3), including antidepressants (71%), stimulants (19%), and anxiolytics (13%). Fifty-four percent of those who did so reported that combining medications with psychedelics had risks. Although most participants believed that the presence of a trained therapist or healthcare professional was important to a successful psychedelic experience, only 15% of participants reported having used psychedelics under such settings (Table 4). Most participants also indicated that they would use psychedelics with the support of either a legal (81%) or underground (71%) therapist if one were available. The most common reasons for not seeking out a healthcare provider in this context were being uncertain of where to find that support (51%) or lack of affordability (28%). Notably, 75%–85% of participants agreed or strongly agreed that therapists or healthcare providers who were offering support during psychedelic experiences should have experience using psychedelics themselves.

**Table 3:** Psychiatric medication use with psychedelics.

Have you ever taken any psychiatric medications on the same day as a psychedelic (*n* = 1,194)	*n* (%)
Yes	283 (23%)
No	911 (74%)
Are there any risks in combining any of these medication(s) with psychedelics? (*n* = 190)	
Yes, there are risks	100 (53%)
No, there are not risks	90 (47%)
Medications used on day of psychedelics (select all that apply) (*n* = 283)	
Antidepressants	200 (71%)
Stimulants	54 (19%)
Nonstimulant ADHD Medications	6 (2%)
Neuroleptics	27 (10%)
Anxiolytics	36 (13%)
Mood Stabilizers/Antiepileptics	36 (13%)
Other CNS Medications	5 (2%)

**Table 4:** Experiences with and attitudes toward taking psychedelics therapeutically.

Have you consumed psychedelics with a trained therapist or healthcare professional?	*n* (%)
Yes	179 (15%)
No	1,042 (85%)
How important is it to have a trained therapist or healthcare professional during the use of psychedelics for therapeutic purposes?	
Not at all important	88 (7%)
Somewhat important	252 (21%)
Moderately important	329 (27%)
Very important	323 (26%)
Extremely important	229 (19%)
Why have not you ever consumed psychedelics under the guidance or support of a trained therapist or healthcare professional? (Select all that apply)	
Not using psychedelics therapeutically	118 (10%)
Did not feel I needed to	241 (20%)
Did not know it existed	238 (19%)
Did not know where/how to find it	620 (51%)
Could not afford it	343 (28%)
Other	154 (13%)
How likely would you be to consume psychedelics under the guidance or support of an “underground” (i.e., not legalized) guide if one were available to you?	
Very unlikely	40 (3%)
Unlikely	80 (7%)
Neither likely nor unlikely	232 (19%)
Likely	425 (35%)
Very likely	443 (36%)
How likely would you be to consume psychedelics under the guidance or support of a trained therapist or healthcare professional if these services were legal and available to you?	
Very unlikely	43 (3%)
Unlikely	47 (4%)
Neither likely nor unlikely	138 (11%)
Likely	311 (26%)
Very likely	682 (56%)
Therapists need to experience psychedelics themselves to properly guide others	n (%)
Strongly disagree	8 (1%)
Disagree	34 (3%)
Neither agree nor disagree	141 (12%)
Agree	347 (28%)
Strongly agree	691 (56%)
Health care professionals need to experience psychedelics themselves to properly integrate psychedelics into their practice (not necessarily as guides during sessions).	n (%)
Strongly disagree	6 (<1%)
Disagree	67 (6%)
Neither agree nor disagree	230 (19%)
Agree	367 (30%)
Strongly agree	551 (45%)

Finally, 70% of participants reported always or often having adequate preparation for a psychedelic experience, 51% reported always or often having adequate support during psychedelic experiences, and 48% reported always or often having adequate support after a psychedelic experience (Table 5). The most common reason people reported not having adequate preparation or support was because they did not need it (35%), were not using psychedelics therapeutically (28%), or did not know where or how to find that support (28%). Those who discussed psychedelics with their primary healthcare provider were more likely to believe that they had adequate preparation for a psychedelic experience (*t*(1096) = 3.65, *p* < 0.001, *d* = 0.27), support during a psychedelic experience (*t*(1095) = 2.89, *p* = 0.002, *d* = 0.21), and support after a psychedelic experience (*t*(1095) = 3.74, *p* < 0.001, *d* = 0.27). There were no differences in perceived preparation adequacy (*t*(272) = 1.55, *p* = 0.001, *d* = 0.19), support during a psychedelic experience (*t*(272) = 0.41, *p* = 0.679, *d* = 0.05), or support after a psychedelic experience (*t*(272) = 0.96, *p* = 0.340, *d* = 0.12), by whether participants with a PsyCP disclosed their psychedelic use.

**Table 5:** Participant perceptions of support surrounding the psychedelic experience.

I have had adequate preparation for a psychedelic experience	*n* (%)
Never	55 (5%)
Rarely	67 (5%)
Sometimes	239 (20%)
Often	474 (39%)
Always	381 (31%)
I have had adequate support during a psychedelic experience	
Never	121 (10%)
Rarely	151 (12%)
Sometimes	324 (27%)
Often	331 (27%)
Always	288 (24%)
I have had adequate support after a psychedelic experience	
Never	144 (12%)
Rarely	193 (16%)
Sometimes	284 (23%)
Often	272 (22%)
Always	322 (26%)
Why do you feel you did not always have adequate preparation and/or support? (Select all that apply)	
Not using psychedelics therapeutically	341 (28%)
Did not feel I needed to	424 (35%)
Did not know it existed	186 (15%)
Did not know where/how to find it	340 (28%)
Could not afford it	119 (10%)
Other:	126 (10%)

## 4. Discussion

To the best of our knowledge, this is the first survey to explore participants’ interactions with their PsyCP around psychedelics. Consistent with our hypothesis, a considerably higher percentage of participants reported discussing psychedelics with their PsyCP compared to their PCP, and participants reported greater confidence in their PsyCP’s ability to integrate psychedelics into clinical care vs. their PCP. However, this confidence was still quite low, and over 20% of participants reported taking potentially interacting psychiatric medications with psychedelics on the same day. Our findings also uncover that while many people desire access to a therapist or guide for their psychedelic experiences, half or more of participants reported adequate preparation before, support during, and integration of their psychedelic experiences. Lastly, we report desired characteristics of healthcare providers who work with patients using psychedelics, which include personal experience with psychedelics and competence in the domain of psychedelic knowledge. Taken together, these findings highlight key areas for future research and policy efforts to enhance safety and provide additional supports for clinicians interacting with patients who use psychedelics.

Disclosure of psychedelics use to PsyCPs was higher than toward PCPs, perhaps afforded by the narrowed scope on mental health and often longer appointments. Still, respondents reported barriers to disclosure, including stigma and legal concerns. This concern is valid as prescription of controlled substances might reasonably be withheld by providers due to concerns about misuse of an illegal drug (i.e., psychedelics). Participants also felt that their care providers were not adequately knowledgeable about psychedelics, which aligns with a 2022 survey that examined psychiatrists’ knowledge of psychedelics and found that the average score was 4.2 ± 1.8 out of a possible 8 points (3). At that time, their most pronounced knowledge gaps were in an overestimation of the prevalence of hallucinogen use disorder and a limited awareness of the current status of psychedelic research.

This low rate of disclosure may affect the safety of psychedelic experiences. Indeed, despite many participants believing there were risks associated with mixing psychedelics and psychiatric medications, 23% of participants reported using psychiatric medications on the same day as a psychedelic, most commonly antidepressants, stimulants, and benzodiazepines. Many medications, especially antidepressants, likely interact with classical psychedelics (26). From a safety standpoint, this is most concerning because of the risk of serotonin syndrome, a potentially serious side effect in which heightened serotonergic activity leads to autonomic instability, neuromuscular changes, and mental status change (27). MDMA has the highest risk of serotonin syndrome among common psychedelic substances (28). Although recent studies show that the risk of serotonin syndrome with psilocybin is relatively low at the usual doses seen with recreational and therapeutic use (27), future research is needed to understand appropriate use of psychedelics in the context of concomitant medications. Indeed, a large systematic review showed numerous potential interactions between MDMA and alpha-2 adrenoceptor agonists, antipsychotics, and antidepressants, and between psilocybin and antipsychotics and anxiolytics (26). Outcomes of interactions may include blunting of psychedelic effects, increased risk of serotonin syndrome, as well as pharmacokinetic and/or pharmacodynamic changes. Taken together, these findings suggest that a nuanced discussion with a provider would provide opportunities to reduce risk and appropriately account for these interactions among people using psychedelics.

Although many respondents endorsed the importance of support from a trained therapist or healthcare professional when using psychedelics for therapeutic purposes, only 15% had consumed psychedelics with such support, largely because they did not know how to find these resources and due to affordability. Most participants agreed or strongly agreed that therapists offering psychedelic-assisted therapy should have personal experience with psychedelics. This poses an interesting challenge to the field, as there are currently legal barriers and professional consequences to providers using psychedelics in the United States, and the personal experience of any therapeutic drug’s effects is generally not part of provider training. However, many psychedelic therapists in clinical trials do report personal use of psychedelics, demonstrated by a survey of psychotherapists associated with Usona Institute’s Phase II clinical trial in which 28 of the 32 (88%) of respondents reported personal experience with at least one serotonergic psychedelic (29).

Notably, whereas 81% reported being likely or very likely to consume psychedelics with a trained therapist or guide, 71% were also open to using an “underground” guide if one were available, indicating substantial comfort with proceeding without sanctioned medical or mental health oversight. However, despite most participants not having support from a trained therapist for their psychedelic experiences, many reported having adequate preparation, support during, and integration after their experience. This suggests that individuals within this population are obtaining relevant information on how to use these substances as well as support from friends and other trusted adults to fill this gap. Compared to those who did not disclose, participants who disclosed psychedelic use to their primary care provider reported small but statistically significantly higher degrees of preparation, support during, and support after their psychedelic experience, suggesting that communication with healthcare providers may be important for creating appropriate set and setting for the psychedelic experience. Given the widespread interest in psychedelics but considerable gaps in availability and accessibility of trained healthcare providers (30), future research should focus on understanding what level of support is needed for people using psychedelics for various medical conditions, as well as patient factors (e.g., co-morbidities) that might require extra support.

### 4.1. Implications

A recent study projected that over half of US States will legalize psychedelics by 2034 to 2037 (1). With this rapidly changing landscape, we believe that psychiatrists, therapists, and PCPs have a critical role to play in facilitating safe use of psychedelics in healthcare. To make best use of therapeutic encounters with patients using psychedelics, providers need access to unbiased and thoughtful education about how to work with patients using these substances. This is especially important for psychotherapists, who will likely be doing the bulk of the clinical work in this area and will be on the front lines of working with patients in the suggestible, vulnerable state elicited by these compounds (19). More research is needed to help properly inform the clinical space, as there remains much uncertainty about what clinical factors predict a therapeutic vs. negative psychedelic experience, what kind of care is necessary prior to and after such experiences, and what therapeutic modalities should be employed to help maximize therapeutic outcomes.

We also believe it critical that providers have sufficient training to develop a trusting therapeutic alliance around psychedelic use, inquire about substance use using nonjudgmental language, and present risks, particularly interaction risks, clearly and at a patient-appropriate level of understanding. A lack of trust for their PsyCP was cited as a barrier to psychedelics discussion for 23% of respondents. Assuming this lack of trust is not limited to divulging information about illegal activities, this is alarming as a poor therapeutic alliance is likely to negatively impact the patient’s care more broadly (31).

A key legal barrier to appropriately addressing psychedelics in healthcare remains the Schedule I status of virtually all psychedelics, excepting ketamine. Given the active study and promising results throughout the peer-reviewed literature, that legal status does not align with the known safety profile and clinical potential of these substances. Accordingly, the expeditious revision of regulations and recommendations that are incongruent with our best understanding of medical reality should be a paramount priority (32).

### 4.2. Limitations and strengths

The present study is limited by the convenience sampling strategy, which recruited via a psychedelics advocacy event and online forums. Further, our findings may not generalize to other populations as the participants were largely White, had an average age of 39, and included many psychedelic enthusiasts and advocates. We also acknowledge that our approach is rooted in a western academic tradition and does not address the indigenous traditions associated with psychedelics, which may provide support and integration in ways that were not interrogated by our questionnaire. We would also expect to see different trends in data with greater ethnic, racial, and socioeconomic diversity based on cultural factors. Finally, we did not investigate provider viewpoints on interactions with people using psychedelics nor did we investigate factors (e.g., diagnoses, psychedelics used) associated with feeling adequately supported during a psychedelic experience—both of which are fruitful areas for future research. These limitations are offset by several strengths, including our large sample size and questions that gauge previously unexamined aspects of naturalistic psychedelic use, including interactions with PCPs and PsyCP, desire for therapist support, and investigation of medications taken concomitantly with psychedelics.

## 5. Conclusion

In this large survey study of individuals using psychedelics naturalistically, we show that participants generally used psychedelics for managing mental health issues and desired more support from therapists or mental health professionals while using psychedelics. Participants engaged with PsyCP more frequently than with PCPs about psychedelic use, although confidence was low in both PsyCP and PCP ability to integrate psychedelics into current treatment. Many participants reported using psychiatric medications on the same day as psychedelics, including medications that may increase risk of side effects or potentially attenuate psychedelic effects (e.g., benzodiazepines, SSRIs). There were significant gaps in access and knowledge around finding either an underground or licensed therapist for such support. As psychedelics become more widely available through rapidly advancing liberalization efforts, our results demonstrate the continued need for healthcare provider education around psychedelics, as well as policies supporting training and harm reduction for those using these substances.

## Data availability statement

The raw data supporting the conclusions of this article will be made available by the authors, without undue reservation.

## Ethics statement

The studies involving humans were approved by Institutional Review Board University of Michigan Medicine. The studies were conducted in accordance with the local legislation and institutional requirements. The participants provided their written informed consent to participate in this study.

## Author contributions

KB, JB, CF, MH, NG, NK, and DK helped to conceive of the study and designed the survey. JB, CF, MH, NG, NK, and DK helped to distribute the survey. DK performed data cleaning and analyses. KB, CW, and KC drafted the manuscript. KB, KC, CW, MH, NG, NK, CF, JB, and DK provided significant feedback and critically revised the manuscript. All authors contributed to the article and approved the submitted version.

## Funding

KB’s effort on this publication was partially supported by the National Institute on Drug Abuse of the National Institutes of Health under K01DA049219. The content is solely the responsibility of the authors and does not necessarily represent the official views of the National Institutes of Health. The authors declare no other funding sources.

## Conflict of interest

NG owns stock in Cybin Inc., Mind Medicine Inc., Numinus Wellness, Revive Therapeutics, Braxia Scientific, and Compass Pathways. CW owns stock in Mind Medicine Inc., Numinus Wellness, and Tryp Therapeutics. KB has received grant funding from Tryp Therapeutics for protocol development and sits on a data safety and monitoring board for an ongoing clinical trial with Vireo Health (unpaid). JB is the Founder/President of the Michigan Psychedelic Society, Co-Founder of Decriminalize Nature Michigan, Executive Director of Decriminalize Nature Ann Arbor, Psychedelic Integration and Music Therapist at Blue Sage Health Consulting.

The remaining authors declare that the research was conducted in the absence of any commercial or financial relationships that could be construed as a potential conflict of interest.

## Publisher’s note

All claims expressed in this article are solely those of the authors and do not necessarily represent those of their affiliated organizations, or those of the publisher, the editors and the reviewers. Any product that may be evaluated in this article, or claim that may be made by its manufacturer, is not guaranteed or endorsed by the publisher.
